# The anterior versus posterior hippocampal oscillations debate in human spatial navigation: evidence from an electrocorticographic case study

**DOI:** 10.1002/brb3.507

**Published:** 2016-06-27

**Authors:** Isabel C. Duarte, João Castelhano, Francisco Sales, Miguel Castelo‐Branco

**Affiliations:** ^1^ Brain Imaging NetworkICNASCoimbraPortugal; ^2^ Institute for Biomedical Imaging and Life SciencesCNC.IBILIUniversity of CoimbraCoimbraPortugal; ^3^ Department of NeurologyCHUCCoimbraPortugal

**Keywords:** Delta, electrocorticography, hippocampus, oscillations, theta

## Abstract

**Introduction:**

Hippocampal oscillations have been regularly described as playing a dominant role in spatial memory and navigation in rodents. In humans, the relative role of anterior versus posterior rhythms during navigational memory is not established.

**Methods:**

Here, we tested this hypothesis using direct brain ECoG recordings in the anterior and posterior hippocampus of a patient, in a navigational task requiring spatial memory. We assessed multiple oscillatory bands during encoding and retrieval phases.

**Results:**

We found navigation related 1–3.5 Hz activity during retrieval, both in the anterior and posterior hippocampus. Activity between 4 and 8 Hz was identified during both encoding and retrieval, only in the anterior hippocampus.

**Conclusions:**

Our findings are consistent with the view that an anterior/posterior functional gradient is present in the hippocampus, and involves two distinct neuronal networks, supporting either encoding or retrieval processes**.** Although this is a single case scenario, these findings suggest that neural oscillations during spatial navigation do vary across hippocampal subregions, as a function of encoding versus retrieval processes during the mnemonic process. In this single case study, the results point to the presence of a dual involvement of multiple frequency bands across hippocampal subregions during encoding and retrieval. Although these results need generalization, they provide a new perspective on distinct physiological properties of the anterior and posterior hippocampus in human spatial navigation during encoding and retrieval.

## Introduction

It is widely believed from rodent studies that theta oscillations, defined as ranging from about 3 to 12 Hz in rodents (Klimesch 1999), have a dominant role during spatial navigation and memory. These rhythms appear reliably in direct electrical recordings from the rodent hippocampus (O'Keefe and Recce 1993; Seager et al. 2002). Oscillatory patterning is increased during successful memory encoding (Seager et al. 2002) and may provide a mechanistic framework for the processing of the rodents’ location in the environment. Path integration seems to occur through phase‐coupling of neural firing with the oscillatory pattern to compute the animal's spatial location during navigation (Burgess et al. 2007).

However, recent studies challenge the view that only particular theta rhythms**,** (defined as ranging from about 4 to 7.5 Hz in humans (Klimesch 1999), are exclusively involved during navigational memory in humans (Ekstrom et al. 2005; Jacobs et al. 2007; Clemens et al. 2013; Watrous et al. 2013; Jacobs 2014), as well as in other mammals, such as the rhesus macaque and bat (Skaggs et al. 2007; Yartsev et al. 2011). When taking into account positive or negative reports on the role of particular oscillatory rhythms in theories of navigation and memory, one has, however, to take into account that bands are often defined according to particular categorical boundaries. Here, we avoided defining arbitrary boundaries and studied navigation‐related oscillations across a continuous array of frequencies.

In rodents, theta activity typically manifests continuously until the cessation of movement (Vanderwolf 1969). In contrast, in humans, when reported, theta appears in short and phasic bursts instead of the persistent activity typically seen in rodents (de Araújo et al. 2002; Bischof and Boulanger 2003; Ekstrom et al. 2005). Ekstrom and colleagues suggested that particular theta bursts that they identified in humans are related to movements and not necessarily only for navigation or spatial memory (Ekstrom et al. 2005).

This sparseness of theta rhythms adds further insights into the studies in rodents, as those oscillatory mechanisms seem to be potentially translated to rhythms occurring at other frequencies that could be relevant for human navigation, as reviewed by Klimesch 1999.

Electrocorticographic (ECoG) studies provide a unique opportunity to study the functional parcellation of the hippocampus in which concerns spatial navigation. A few of such studies have suggested that the human hippocampus also exhibits slower oscillations in the frequency range 1–4 Hz (the delta band is categorically defined between 1.5 and 3.5 according to Klimesch (1999)) during spatial memory and navigational computerized tasks (Jacobs et al. 2007; Clemens et al. 2013). Jacobs et al. (2007)analyzed spike linked oscillations during a car driver game and identified hippocampal 1–4 Hz oscillations, as well as gamma oscillations (35–80 Hz) that were task‐related. Clemens and colleagues also confirmed in an ECoG study, that the engagement in a navigational task resulted in increased activity in the 1–4 Hz band (Clemens et al. 2013).

Jacobs recently suggested that the human analog of the rodents’ theta rhythm is actually oscillating at lower frequencies (Jacobs 2014). Furthermore, Zhang and Jacobs actually refer to the human hippocampal oscillations between 2 and 10 Hz as “theta” oscillations (Zhang and Jacobs 2015), although we agree that these definitions are arbitrary, and that some of these bands might actually be defined as low alpha activity.

Together, the electrophysiological studies in rodents and in humans show that neural oscillations in hippocampus play a critical role in memory formation, irrespective of putative frequency differences. It is not known whether these differences are related to different cortical architectures across species. Moreover, they might also be related with the well‐known human hemispheric specialization in terms of visual and verbal material or its long‐axis (anterior‐posterior) specialization. Concerning this axis, we recently found in a functional MRI study a dichotomy between anterior and posterior hippocampus during navigational memory, suggestive of the existence of antagonistic coupling between these structures (Duarte et al. 2014). A few structural studies on navigational memory had also suggested before that anterior and posterior most hippocampal regions are associated with differential and antagonistic neural processing (Maguire et al. 2000; Leporé et al. 2009). One example, is the famous anatomical study in taxi drivers, whereby posterior enlargement was found at the cost of an anterior decrease (Maguire et al. 2000), and the mirror symmetric findings observed in blind people, in whom the right anterior hippocampus was found to be larger, at the cost of a smaller posterior hippocampus, as compared with sighted people (Leporé et al. 2009). More recent studies suggest a model for an anterior/posterior gradient inside the hippocampus (Kim, 2015), whereby its posterior portion is related to internal attention and the default mode network, whereas its anterior portion is related to external attention and the dorsal attention network (Robinson et al., 2015).

Given the unique opportunity to use direct brain ECoG recordings, we tested spatial memory using the same paradigm as in a previous fMRI study (Duarte et al. 2014) in an epileptic patient with electrodes surgically implanted deep in the left hippocampus. We did not focus on a specific oscillatory band, since the human hippocampus exhibits different oscillatory patterns as compared to rodents (Zhang and Jacobs 2015). While one of such bands could potentially represent a functional analog of the rodents’ theta oscillations, the main goal of this study was rather focused on the distinct functional properties of the anterior and posterior hippocampus.

## Methods

### Participant

This study included an epileptic patient who underwent presurgical ECoG evaluation. ECoG is a standard clinical procedure for patients with severe and refractory epilepsy proposed to resective surgery. The procedure involves having surgically implanted electrodes during several days to map the seizure onset region. The study was approved by the Ethics Committee of Faculty of Medicine of University of Coimbra to be performed after clinical procedures and under the patient's written informed consent, in accordance with the Declaration of Helsinki.

The patient was a 31‐year‐old male. He was right‐handed and had normal vision. Neurological examination revealed well preserved verbal and visual memory. During the monitoring, the antiepileptic medication was interrupted. It included daily doses of 2500 mg of valproic acid, 1200 mg of carbamazepine, 300 mg of zonisamide and 2000 mg of levetiracetam. The subject performed the navigation task during hospitalization at his free time, after standard clinical procedures, and in the presence of the specialist team who regularly accompanied the monitoring.

### Task

The navigational memory task was used before in an fMRI study (for details, see (Duarte et al. 2014). The paradigm comprised a boxcar‐based design, consisting in 14 pairs of encoding/retrieval blocks of 21 sec. Each encoding block is followed by the respective retrieval block, and the interval between them is jittered and can last 3, 6 or 9 sec. A baseline period occurs between each pair of encoding/retrieval and it lasts 9 sec. Each encoding/retrieval pair is defined by a different 3D office‐like scenario, with a specific set of chairs and tables, different of any other to minimize confusion with anterior scenes. The target chairs, which position the subject has to memorize, are presented only in the encoding phase. The door and the picture in the wall are equal in all scenes and therefore their positions remain the same along the task. The virtual environments were created using the virtual‐reality toolkit Vizard (WorldViz, Santa Barbara, CA).

During encoding, the subject was instructed to navigate toward the door and along the path to click in the targets as he memorizes their position. During the retrieval, he had to mark the missing objects’ position. In half of the retrieval scenes, the starting position and the angle of vision in the retrieval phase was different from the ones in the encoding phase to preclude the subject to restore the same frame of reference, as we did in previous studies (Bernardino et al. 2013; Duarte et al. 2014). No changes were implemented, as required, in the egocentric retrieval. In sum, half of the retrieval blocks were performed requiring egocentric representations and the other half required the use of allocentric representations.

The subject performed the computerized task at his bed and seeing the virtual scenario in the LCD of his room. Active navigation was enabled by a joystick.

The subject had the opportunity to train and acquaint himself with the paradigm and the navigation with the joystick. The paradigm was kept as simple as possible, since a complex paradigm could potentially recruit novelty‐related responses in the anterior hippocampus (Hashimoto et al. 2012). No major changes in the navigation speed were allowed, to avoid the confound suggested by a study in rodents pointing that theta oscillations correlates with the movement (Sławińska and Kasicki 1998). Measures of performance were obtained as described earlier (Duarte et al. 2014) and confirmed that the subject was fully engaged in the navigational memory task.

### Data acquisition and analysis

**Figure 1:**
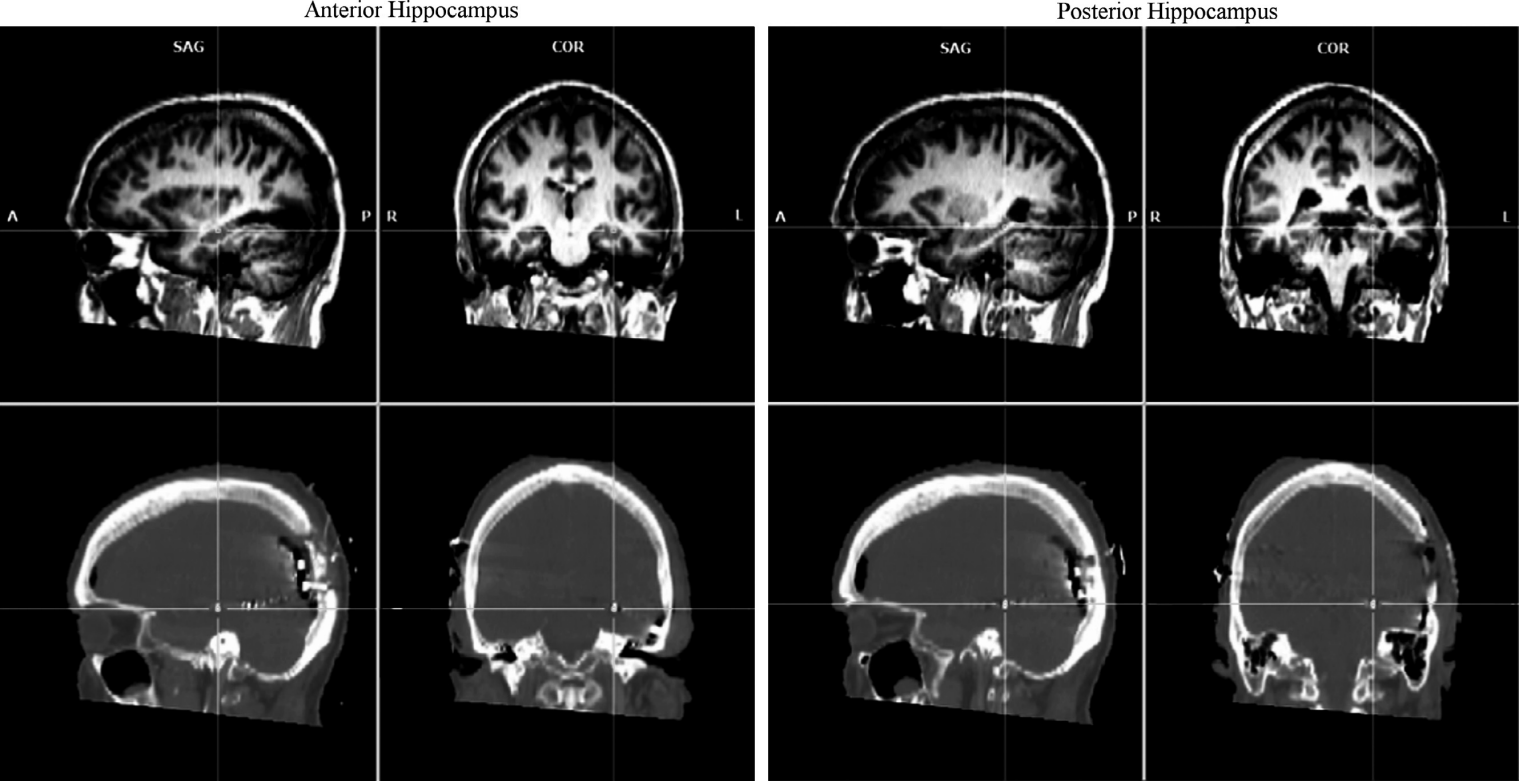
Sagittal (left) and coronal (right) views of MRI (top) and CT (bottom) images of the electrodes position in the anterior and posterior hippocampus.

Electrocorticographic signal was acquired at the Coimbra University Hospital. The signal from 48 electrodes was recorded at high sampling rate (5 kHz), low pass filter at 500 Hz, A/D accuracy of 2.80 *μ*V through a SynAmps2/RT amplifier, using reference electrodes in the right scalp and using the module Maglink RT Acquire in the Scan 4.5 software (NeuroScan, Compumedics, Charlotte, NC). The continuous data were digitally filtered between 1 and 500 Hz and referencing was done to the average reference, as described elsewhere (Zhang and Jacobs 2015), using Scan 4.5. Further processing was performed in the EEGlab toolbox (Delorme and Makeig 2004) for MATlab (MathWorks, Natick, MA). Epochs from −3000 to 21,000 msec were obtained locked to the stimuli onset. Epoch rejection was done by visual inspection to ensure the data was free of ictal artifacts. For encoding, 252 (out of 294) seconds were included in the analysis. For retrieval, 231 sec of task were considered free of artifacts by visual inspection and were included. The time‐frequency analysis (−2000–5000 msec) included frequencies between 1 and 200 Hz, baseline including all negative time‐points, and significance level of 0.01 (number of permutation replications to accumulate: 200). Time‐frequency decomposition done using the function *pop_newtimef()*, with the wavelet beginning with a 3‐cycle, increasing linearly with the frequency. Correction for multiple comparisons was done using false detection rate (*α* = 0.004785). Given the aim of the study, we focused on the medial temporal lobe electrodes 36, in the anterior hippocampus (Talairach coordinates *x*,*y*,*z* = ‐31,‐19,‐10), and 39, near the posterior hippocampus (*x*,*y*,*z* = ‐30,‐36,‐8) (Fig. 1).

To perform the statistical analysis between conditions and electrodes, we computed single trial power in subepochs set to 3 sec (each block of 21 sec was divided into seven smaller epochs), maintaining the preblock baseline. The time/frequency decomposition was computed with a frequency resolution of 0.2 Hz. The norm and the normalization to the same baseline was calculated and the results were presented scaled to decibels (10 × log10).

## Results

**Figure 2:**
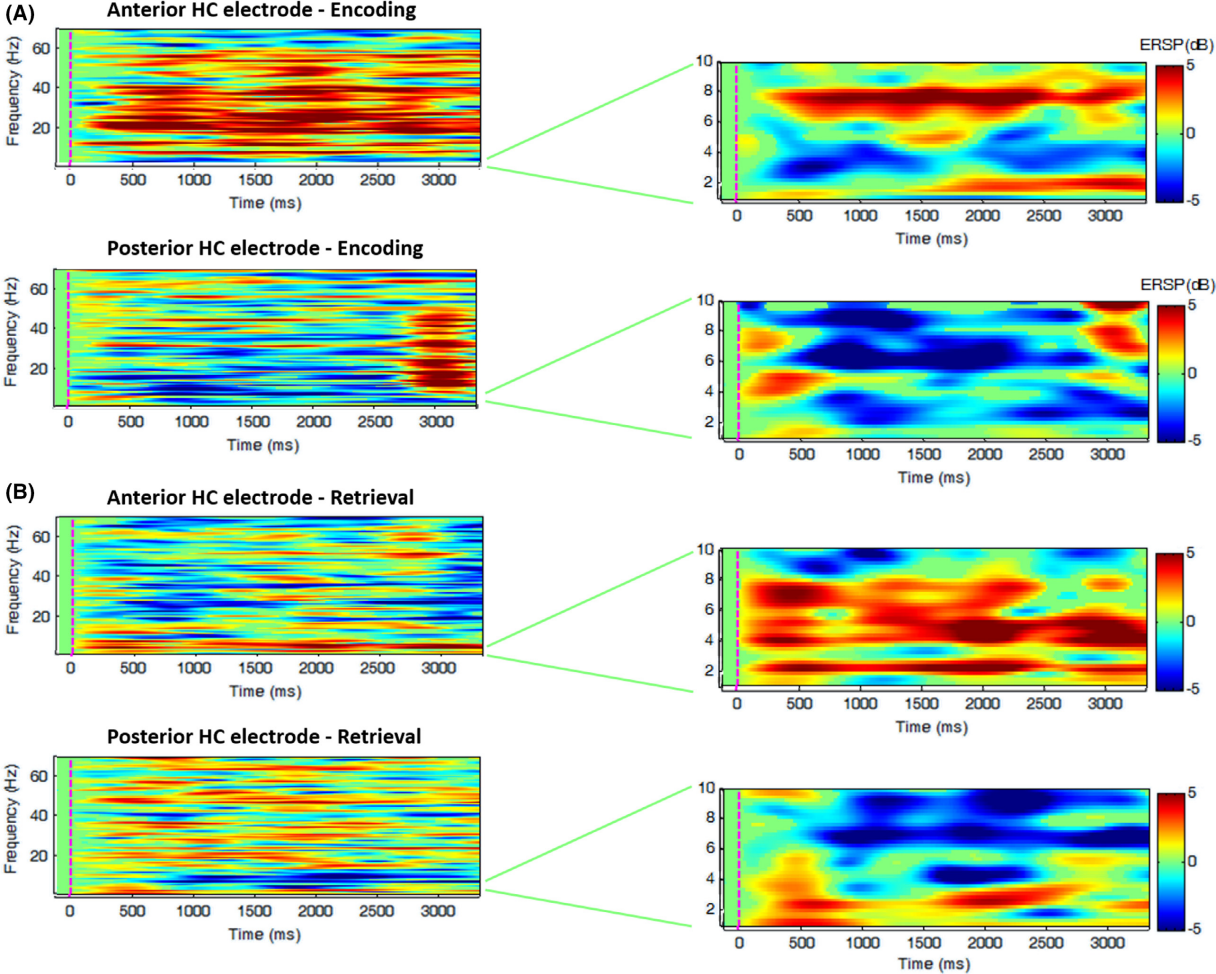
Time‐frequency analysis in the anterior and posterior hippocampus during (A) encoding and (B) retrieval (*α* = 0.01, nonsignificant output values are plotted in green).

Interesting changes in relation to the baseline condition, at the 0.01 significance level, were found in lower frequencies between 2 and 10 Hz and in the gamma band. We found that slower activity between 1 and 3.5 Hz was present during retrieval and that activity between 4 and 8 Hz was only present in the anterior hippocampus during both encoding and retrieval (Fig. 2).

Accordingly, during the *encoding* phase, the anterior hippocampus showed decreased activity in the 2–4 Hz band and increased activity around the 4–8 Hz band, as well as increases in activity in frequencies up to 50 Hz. At the same time, the posterior region showed significant decreases in both frequency intervals, from 1 up to 10 Hz.

During the *retrieval* phase, we found activity increases in the 1.5–3 Hz and 4–8 Hz frequency bands in the anterior electrode. At the same time, the posterior one showed activity increases in the 1–3.5 Hz band and decreases in the 4–10 Hz band. No differences above those bands were found during retrieval.

### Anterior versus posterior comparisons

Time‐frequency analysis in the anterior and posterior hippocampus showed opposite effects between both electrodes in the 5–8 Hz band, especially during encoding. In this range, higher activity was found in the anterior hippocampus, whereas a lower activity was found in its posterior portion.

### Encoding versus retrieval

During *retrieval*, we found activity increases in the 1–3 Hz band, in contrast with the decreases found during *encoding* in a similar band**,** in both anterior and posterior positions.

**Figure 3:**
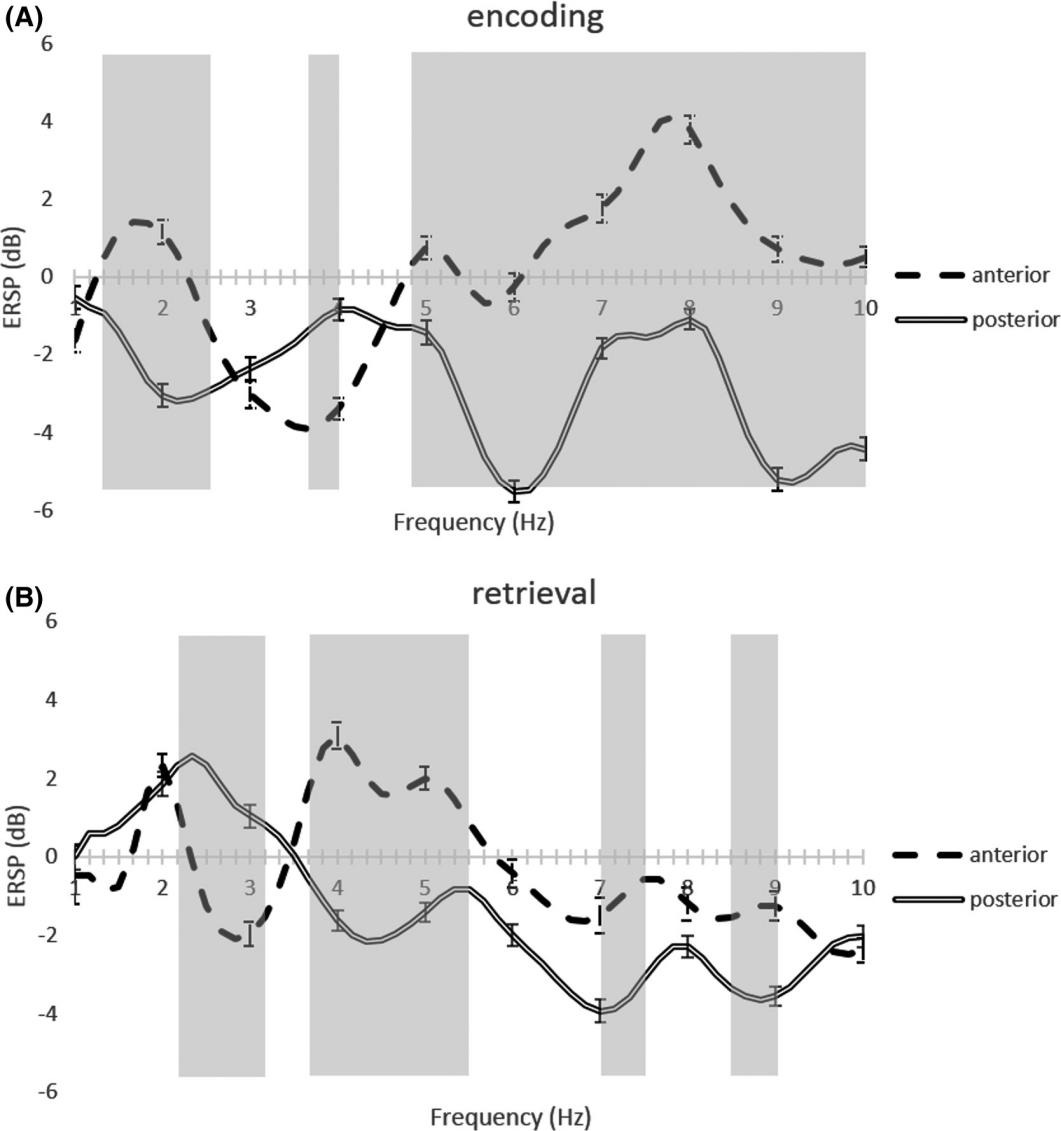
Plot of the averaged event‐related spectral perturbation value for each frequency in the delta and theta band and the respective standard error in the anterior and posterior Hippocampus during (A) encoding and (B) retrieval. Gray regions stand for statistical different values of ERSP (*P* < 0.0001).

To show the consistency of these results along the whole block, we plotted the averaged event‐related spectral perturbation, ERSP, of the computed power of the 3 sec subepochs and the respective standard error (Fig. 3). Consistent results were found for each frequency, strengthening the differential pattern of anterior/posterior findings in the 5–8 Hz band. Statistically different frequencies are marked with gray color in the background in Figure 3 (resultant from a paired *t*‐test with *P* < 0.0001).

We also tested the opposite encoding/retrieval effects in the lower frequency regions. In the anterior hippocampus, significant deactivation/activation differences (t(76) = 7.89; *P* < 0.000001) were found at 3 Hz when comparing encoding (mean = −3.42; SD = 2.58) and retrieval (mean = −0.23; SD = 2.51). In the posterior hippocampus, encoding (mean = −3.47; SD = 2.45) and retrieval (mean = 1.00; SD = 2.06) at 3 Hz also showed significant differences (t(76) = 14.31; *P* < 0.000001).

## Discussion

A broad range of rhythms in anterior and posterior hippocampus may be relevant in navigational memory.

The complex pattern found on this subject using this paradigm points to the presence of an involvement of multiple frequency subbands across hippocampal subregions in this patient. We found specific oscillatory patterns in the 1.5–8 Hz region according to each subhippocampal area (anterior or posterior) and the cognitive process (encoding or retrieval). Our findings suggest that we should have consideration to the mnemonic process (encoding or retrieval) under assessment and to the hippocampal subregions when studying the roles neural oscillations during spatial navigation in humans.

### Distinct oscillatory patterns between encoding and retrieval

We found an antagonistic pattern in the 1.5–3 Hz band between encoding and retrieval, in both anterior and posterior regions. Higher activity in this band was found for retrieval blocks, whereas lower activity was found during the encoding period. Conversely, higher modulation in frequencies up to 50 Hz was found in the anterior hippocampus during the encoding periods.

Jacobs et al. (2007) and Clemens et al. (2013) results showed increased activity in the 1–4 Hz band while the subjects where engaged in navigational tasks. Clemens refers also a higher modulation in this frequency region during recall than during the encoding (Clemens et al. 2013). Jacobs’ paradigm design does not allow for the discrimination between encoding and retrieval phase, however, they also report the finding of hippocampal oscillations at 1–4 Hz and increases in gamma oscillatory activity during the task (Jacobs et al. 2007). Robinson and colleagues (Robinson et al., 2015) suggest that encoding and retrieval are distinct processes in their nature, requiring either so‐called external or internal attention processes, respectively. Therefore, the two different cognitive processes imply the involvement of two distinct neuronal networks: the dorsal attention network during encoding and the default mode network during retrieval (Robinson et al., 2015). We think that our results can meet this view**,** within the limitations of a single subject study, due to the use of a paradigm that separates encoding from retrieval.

### Further evidence for anterior/posterior functional parcellation

We had observed in a previous fMRI study that the anterior and posterior hippocampus bears a functional dichotomy during navigational memory, which is now corroborated by direct recordings. This long‐axis functional dichotomy is becoming increasingly accepted as it is detailed in recent works in fMRI and DTI (Kim, 2015; Robinson et al., 2015). Moreover, recently in a study using deep electrodes implanted along the hippocampal axis, Zhang and Jacobs’ results suggest the presence of traveling theta oscillations similar to that discovery in rodents, moving from posterior to anterior hippocampal subregions (Zhang and Jacobs 2015). If the mnemonic mechanism is similar in humans to what happens in the rodents (Patel et al. 2012), the theta phase is a key factor in the way that hippocampal neurons signal an event. Note that in that work, human theta has been considered to encompass a range between 2 and 10 Hz (Zhang and Jacobs 2015).

The results in this case report showed anteroposterior antagonistic patterns in the 5–8 Hz band, as well as in higher frequencies up to 50 Hz. The anterior electrode presented greater modulation in the 5–8 Hz, during both phases, in contrast with the posterior electrode.

The oscillatory pattern found in this patient also evidences a tight antagonistic coupling between anterior and posterior hippocampus. These opposite patterns of neuronal activity lead to the interesting speculation that, if sustained, they might lead to distinct spatial patterns of long‐term plasticity (Maguire et al. 2000; Leporé et al. 2009).

## Conclusions

The results suggest that consideration should be taken to the mnemonic process (encoding or retrieval) and to the hippocampal subregions when studying the roles of neural oscillations during spatial navigation in humans.

In sum, we found different oscillatory patterning between encoding and retrieval. Slower activity at 1.5–3 Hz was present only during retrieval, both in anterior and posterior hippocampus, and 5–8 Hz activity was present during both encoding and retrieval, however, only in the anterior hippocampus. Even given the high sensitivity and signal‐to‐noise ratio of ECoG recordings, the results of this single case study need to be replicated in a larger number of subjects. A first level approach such as provided in this study, providing significant statistics at the single subject level, should be used in future meta‐analytic approaches, given the difficulty of single ECoG studies – in particular comparing activity between anterior and posterior hippocampus – to have enough sample size to provide generalization to the population.

We hope this work can shed new light by making clear that it is important to separate the cognitive process of navigational memory in its encoding and retrieval phases, and that one should not conceptualize the hippocampus as a single indivisible structure, but rather consider an anterior‐posterior functional gradient inside hippocampus.

## Conflict of Interest

None declared.
